# Distribution and Stability of Polyphenols in Juices Made from Traditional Apple Cultivars Grown in Bosnia and Herzegovina

**DOI:** 10.3390/molecules28010230

**Published:** 2022-12-27

**Authors:** Amila Oras, Asima Akagić, Nermina Spaho, Fuad Gaši, Sanja Oručević Žuljević, Mekjell Meland

**Affiliations:** 1 Faculty of Agriculture and Food Sciences, University of Sarajevo, Zmaja od Bosne 8, 71 000 Sarajevo, Bosnia and Herzegovina; 2 Department of Horticulture, Norwegian Institute of Bioeconomy Research, NIBIO Ullensvang, Ullensvangvegen 1005, NO-5781 Lofthus, Norway

**Keywords:** cloudy and clear juices, peel and pulp, L-ascorbic acid, flavan 3-ols, chlorogenic acid

## Abstract

The present research was undertaken to investigate polyphenolic profiles of peel, pulp and juices made from two standard commercial and five traditional apple cultivars from Bosnia and Herzegovina. The main goal of the study was to monitor the distribution and changes of polyphenolic profiles through different phases of apples’ processing into cloudy and clear juices, with regard to L-ascorbic acid pretreatment. Quantitative determination of phenolic compounds was carried out by using high-performance liquid chromatography with diode-array detection. The obtained results showed that traditional cultivars, namely ‘Paradija’ and ‘Prijedorska zelenika’, displayed significantly higher content of these compounds compared to commercial ones. Flavan 3-ols and flavonol glycosides were mostly found in peels of all cultivars (21.2–44.1 and 5.40–33.3%, respectively), while phenolic acids along with flavan 3-ols were predominant in the pulp (8.20–30.8 and 5.10–13.9%, respectively). Apples’ processing into juices caused decrease (more than 90%) in the content of all polyphenols and the distribution of these compounds from fruits to final products had a negative trend, particularly evident in clear juices. The most drastic loss occurred in the flavonol glycosides and dihydrochalcones content, while chlorogenic acid displayed quite stable distribution from apples to final products due to its good solubility. Apple mash pretreatment with L-ascorbic acid had a positive impact on the preservation and retention of polyphenols.

## 1. Introduction

Current trends to meet market demands are directed to production of high-quality food products characterized by health-improving potential and added value. These requirements can be met through the processing of raw materials that carry desirable nutritional, bioactive, and sensory properties. Apple (*Malus domestica* Borkh.) is a widely distributed fruit all over the world. It is constantly drawing attention for its rich polyphenolic profile and strong antioxidant capacity [1]. Existing literature evidences have authenticated the linkage between apple and its products’ consumption with reduced risk of cardiovascular diseases, certain cancer types, diabetes, and others [2,3,4,5,6]. Four major health promoting phenolic groups found in most apples are phenolic acids, flavan 3-ols, dihydrochalcones, and flavonol glycosides [7]. In addition to the health benefits, these compounds contribute to apple and juices’ sensory properties such as color, bitterness, and astringency [8,9].

The content of polyphenols in apples is dependent on numerous factors, such as the cultivar, part of fruit, applied agronomic measures, climate conditions, maturity stage, harvesting, and method of processing [10,11,12]. In the terms of fruit, peel contains significantly more polyphenols compared to pulp [13,14]. Moreover, differences between traditional and standard commercial cultivars can be made [15]. Bosnia and Herzegovina (B&H) is well known for its traditional apple cultivars. Those cultivars poses a remarkable nutritional and bioactive properties as well as outstanding sensory characteristics [16,17]. As Alihodzic et al. [18] stated, autochthonous B&H apples have a unique flavor that offers added value and a great opportunity for the fruit processing industry. In addition, they contribute to biodiversity preservation and cherishing of cultural–historical heritage. Various studies have shown that traditional B&H cultivars contain higher polyphenols content in comparison to commercial fruits [19,20]. 

Among all influencing factors, the method of fruit processing is of crucial importance for the polyphenols content, which is particularly important in the juices’ production. Each fruit cell wall disintegration (cutting, grinding, etc.) leads to a decrease in the polyphenols content due to enzymatic oxidation by oxidoreductive enzymes which results in formation of brown pigments [21,22]. Flavanol monomers, hydroxycinnamic acids, and dihydrochalcones are significant contributors in the formation of oxidation products and color of apple juices [23]. There are some reports claiming that chlorogenic acid, catechin, epicatechin, and quercetin are preferred substrates for enzymatic browning [24,25,26]. However, these compounds can also act as inhibitors of enzymatic browning reactions through different mechanisms [27]. As Arnold and Gramza–Michalowska [28] stated, every apple cultivar differs in chemical (phenolic) composition and thus enzymatic browning activities as well. Therefore, apple cultivar selection is marked as a crucial point to be considered by the industries in prevention of intensive enzymatic browning. Polyphenols content decrease trend during the production of juices may be diminished by the addition of L-ascorbic acid as a strong antioxidant which delays enzymatic browning [29]. More recently, riboflavin (vitamin B2) was found to have antibrowning effects as well [30]. However, the appliance of thermal treatment [31] and clarification process (depectinization, clearing, and filtration), which occurs only in the production of clear juices, lead to greater oxidation, degradation, and final removal of these valuable compounds [32,33]. 

In the past, the domestic market was dominated by clear apple juices, while today the number of small processing plants (niche markets) that almost exclusively produce cloudy apple juices is constantly increasing. However, in the production of cloudy and clear juices they primarily use commercial apple cultivars, which are quantitatively more available on the B&H market [34]. These cultivars often lack in required antioxidant potential and sensory attributes such as aroma. On the other hand, traditional apple cultivars in Bosnia and Herzegovina are valuable sources of desirable characteristics and they could be used to enrich juices obtained from commercial cultivars [35]. The main goal of the study is focused on the analysis of the distribution and stability of polyphenolic components of apples and their juices (clear and cloudy), depending on the cultivar, applied pretreatment (L-ascorbic addition), and juice production phase. 

## 2. Results and Discussion

### 2.1. Determination of Polyphenolic Compounds in Apples

The contents of polyphenols in different parts of traditional and commercial apple cultivars are presented in Table 1.

The total polyphenol content in whole fruit ranged from 571.4 mg kg^−1^ of FW (‘Granny Smith’) to 1315.3 mg kg^−1^ of FW (‘Paradija’). In general, it is evident that traditional apple cultivars had significantly higher polyphenols content in comparison to commercial ones, which is in accordance with results reported by Jakobek et al. [36] and Lončarić et al. [37]. One of the reasons for the lower content of polyphenols in commercial apple cultivars is that targeted breeding had led to reduction of these compounds in order to mitigate enzymatic browning and astringent taste [20]. Along with high antioxidant potential, pomological properties, fruit quality, and resistance to abiotic and biotic factors, the main goal for preservation of traditional apple cultivars is the prevention of their possible disappearance and the preservation of biological apple biodiversity.

In the present study, four classes of polyphenols were quantified in all cultivars: phenolic acids, flavan 3-ols, dihydrochalcones, and flavonol glycosides. Among them, phenolic acids and flavan 3-ols were predominant. Traditional cultivars contained mainly flavan 3-ols (‘Funtača’, ‘Rebrača’, ‘Paradija’), while commercial apples were more characterized by phenolic acids content. As Jakobek and Barron [38] reported, old and new apple cultivars differ on the basis of polyphenolic groups and thus can be classified according to abundant proportion of phenolic acids and flavan 3-ols. Statistical analysis revealed significant differences in polyphenols content between peel and pulp. Apple peel of all cultivars contained higher polyphenols content (up to four times, Table 2) compared to pulp, which is consistent with results reported by Preti and Tarola [39] and Illiano et al. [40]. According to Bohinc et al. [41], polyphenols are mostly found in peel since they act like protectors of fruit pulp from different environmental stressors, such as light, heat, insect attacks, etc. The peel of analyzed cultivars mainly contained (Table 2) flavan 3-ols (21.2–44.1%), followed by flavonol glycosides (5.40–33.3%) and phenolic acids (8.10–19.5%), while the content of dihydrochalcones was the lowest (7.0–18.3%). Phenolic groups detected in pulp had the following order: phenolic acids (8.20–30.8%) > flavan 3-ols (5.10–13.9%) > dihydrochalcones (1.0–1.90%) > flavonol glycosides (0.09–0.40%). The polyphenolic profiles obtained in the present study are in consistence with results reported by other authors [42,43]. Phenolic acids, detected in both peel and pulp, included chlorogenic, caffeic, gallic, protocatechuic, and sinapic acid. The content of chlorogenic acid was the highest (50–217.6 mg kg^−1^ FW in peel and 71.8–389 mg kg^−1^ FW in pulp), while caffeic acid was quantified in small amounts. It is generally considered that hydroxycinnamic acids, mainly chlorogenic acid, have a strong influence on the oxidation process and unwanted change of color during the juices production [24]. However, according to Santana–Galvez et al. [44], chlorogenic acid has shown many health-promoting properties, including antioxidant, antimicrobial, anti-inflammatory, and prebiotic activities. In addition, these authors suggest that chlorogenic acid should be used for the formulation of functional food supplements due to its outstanding properties. Traditional cultivar ‘Prijedorska zelenika’ was found to be the richest in chlorogenic acid content. Flavan 3-ols (catechin, epicatechin, procyanidin B1, and procyanidin B2) were mostly abundant in the peels of all analyzed cultivars. Epicatechin and procyanidin B2 contents in peels were the highest (74.8–302.1 and 87.2–149 mg kg^−1^ FW, respectively).

Traditional apples ‘Paradija’, ‘Rebrača’, and ‘Tetovka’ were characterized by high amounts of procyanidin B2, epicatechin, and catechin. Similar results were reported by Gotal et al. [45]. According to Yu et al. [46], procyanidin B2 and epicatechin have various positive health effects, particularly reflected in their antidiabetic properites. Moreover, catechin is a phenolic compound with remarkable antioxidant, antibacterial, antitumor, and anti-inflammatory properties [47,48]. In addition to health promoting attributes, the listed traditional cultivars and their juices posess extraordinary and highly desirable sensory properties as well [35]; therefore, they should be more utilized on an industrial scale. Dihydrochalcones, phloretin and phloridzin, are unique polyphenols found exclusively in apples [49]. The results from this study revealed that the peel of traditional cultivars (‘Tetovka’ and ‘Paradija’) contained higher amounts of dihydrochalcones, peculiarly phloridzin, when compared to commercial cultivars. This finding is of great importance, since phloridzin seems to have promising health benefits, especially in diabetes type 2 treatment [50,51]. Flavonol glycosides (quercetin 3-*O*-glucoside, galactoside, rhamnoside and rutinoside) were mainly present in peel, while in pulp their content was low or even under the treshold of detection (quercetin 3-*O*-rutinoside). As Wang et al. [52] reported, rutin is mainly present in the apple peel. Quercetin 3-*O*-galactoside and quercetin 3-*O*-rhamnoside were leading compounds among flavonol glycosides. The highest contents of quercetin 3-*O*-galactoside and quercetin 3-*O*-rhamnoside were quantified in the peel of cultivar ‘Idared’ (174.2 and 111 mg kg^−1^ of FW, respectively). It is well known that quercetin and its glycosides are powerful dietary antioxidants [53]. As reported by Zymone et al. [54], antioxidant potential of different polyphenolic classes has the following order: flavan 3-ols > flavonols > chalcones > flavones > flavanones > isoflavones. However, there are studies which reported that quercetin has higher potential than certain flavan 3-ols. The credits for quercetin’s health promoting benefits were particularly given in the past two years, when these polyphenols were recognized as powerful compounds in COVID-19 treatment [55]. On the other hand, flavonol glycosides are characterized by limited aqueous solubility, enzymatic degradation, instability, and low bioavailability as well [56].

### 2.2. Distribution of Polyphenols during Juices Production

Polyphenols in apple juices have various health promoting properties [57] and contribute to sensory characteristics of product [58,59]. Along with apple cultivar itself, the processing method has the greatest impact on the content and retention of polyphenols in juice [60]. As expected, processing apples into juices caused evident loss of polyphenols (Table 3). 

The degradation of these bioactive compounds started with apple fruits disintegration during grinding. However, degradation was slightly mitigated in juices with added L-ascorbic acid, as confirmed by statistical analysis of variance. In the research of Mieszczajowska–Frac et al. [61], the amount of L-ascorbic acid added at the beginning (200 mg kg^−1^) was completely oxidized during juice production. In addition, juices with added L-ascorbic acid also had greater turbidity. Ozoglu and Bayindirli [62] and Jang and Moon [63] stated that the effect of L-ascorbic acid is temporary and that it is completely oxidized, so *o*-quinones can accumulate, which leads to the formation of a brown color. Novel techniques for enzyme inactivation and final juice quality preservation include utilization of alternative nonthermal technologies, such as high-pressure carbon dioxide application [64]. As reported by Le Bourvellec et al. [65], despite the addition of L-ascorbic acid, losses of hydroxycinnamic acids and procyanidins were recorded during apple processing. In this study, the total loss of polyphenolic components varied in the range 28.8–48.2% in mashes with added L-ascorbic acid and 35.6–54.4% in mashes without the listed antioxidant. 

The content of phenolic acids was particularly decreased, which was mainly caused by the complete loss of gallic, caffeic, sinapic, and protocatechuic acids. with these phenolic acids, the loss of glucoside-3-*O*-rutinoside, phloretin, and a significant decrease in catechin content was observed. As reported by Jakobek et al. [24] and Serra et al. [26], catechin and quercetin stand for the preferred substrates for polyphenol oxidase (PPO) enzymes oxidation. Although the possible increase of dihydrochalcone phloridzin in mashes was expected due to the crushing of apple seeds which are rich in this component [66], grinding phase did not contribute to this scenario. Flavonol glycosides were rapidly lost during fruit disintegration and accounted for only 30.8–48.9% (mashes without L-ascorbic acid) and 36.6–55.2% (mashes with added L-ascorbic acid) of their initial value. This finding speaks in favor of the fact that they are easily degradable and very unstable, as reported by Renard et al. [67].

The next phases, mashing enzymatic treatment followed by pressing, were crucial in regard of polyphenols retention in apple juices. Pectolytic enzymes, which are added to the mash in order to increase the yield, nutritive and non-nutritive components in the first place, disrupt the barriers of cell walls which makes juice as a substrate for PPO enzymes more accessible for the continuation of enzymatic browning reactions. As stated by some authors [61], enzymatic treatment of mash has a negative impact on the content of polyphenolic components and leads to their loss in the amount of 12–31%. Furthermore, differences in the solubility of certain polyphenolic components determine their greater or lesser distribution in the liquid phase, i.e., juice. For example, chlorogenic acid is the most soluble polyphenolic compound in water [60], so it is not surprising its highest transfer to obtained juices. In addition, this phenolic acid was most abundant in the apples pulp, which is one of the reasons for its greater presence in the raw juice, since the pulp makes up almost 90% of the apple fruit.

The constitution of the apple fruit was also one of the significant reasons for the loss of polyphenols during juicing. Apple peel, in which most of the flavonol glycosides, dihydrochalcones, and flavan-3-ols are concentrated, makes up only 10% of the total fruit. As stated by Ceymann [68] and Brahem et al. [69], phenolic acids are present in juices in high concentration because they are present in the pulp of the apple and are characterized by good solubility in water, while flavonols are primarily located in the skin of the apple and have low solubility in water. Bearing in mind that the peel in the mash is represented in such a small proportion, and that it completely lags behind after pressing the mash, then a significant loss of flavonols in the first place is inevitable. Fruit biomass that remains after pressing is called apple pomace and it is mainly consisted of cell wall polysaccharides and polyphenols that are recognized as functional components important for human health [14]. Thus, with the increasing awareness of sustainability principle in food industry, apple pomace becomes valuable material for utilization in other foods production. According to numerous studies, apple pomace has been recognized as a suitable raw material for enriching confectionery products made from flour in terms of increasing the content of bioactive components and improving sensory characteristics [70]. In general, 74.1–88.1% of total phenols were lost after pressing mashes without L-ascorbic acid pretreatment and slightly less in those with added L-ascorbic acid (71.5–83.9%), which accounts for almost double loss compared to those from the previous phase. 

Colloidal particles of pectin and xylan cause cloudiness of raw and cloudy apple juices, as well as proteins, hemicellulose, and solubilized starch [29]. Polyphenolic compounds can contribute to the turbidity by forming bonds with polysaccharides originating from cell walls. Clarification operations (depectinization by pectinase and xylanase, clearing and filtration) are applied only in the production of clear juices [71]. These operations lead to further loss of polyphenols because of the binding to finning agents and mechanical removal during filtration. Although depectinization phase generally led to further loss of polyphenolic components (71.4–90.1% in CLAA and 74.9–92.9% in CL), it also caused an increase in the content of flavan 3-ols. Considering that flavan-3-ols are often found in the fruit matrix in oligomeric forms (tannins), and that they can form complexes with other structural substances such as pectin, with the hydrolysis of pectin substances by pectolytic enzymes, destruction of their bonds occurs. Their hydrolysis also increases the content of monomeric forms (catechin, epicatechin) that could be quantified. On the other hand, loss of chlorogenic acid was pronounced. As Jen [72] explains, during enzymatic clarification, hydrolysis of chlorogenic acid occurs due to the presence and activity of esterase found in pectolitic mix which is added to depectinize raw juice. Previously, there were proposals that the content of chlorogenic acid in apple juices should be taken as a relevant indicator when evaluating their authenticity, but due to the marked instability of chlorogenic acid during mash blanching and depectinization, these proposals did not come alive. Residual colloidal particles that cause turbidity were removed with clarification and filtration. Their removal in this phase also resulted in the loss of a significant part of the polyphenolic components (81.4–94.8% CL and 81.5–92.2% CLAA). As stated by Duda–Chodak et al. [73], procyanidins are removed during the clarification process in the production of clear juices. They stated that epicatechin and procyanidins are oxidized in this phase, and form high molecular polymers that are absorbed by gelatin, and are also removed during the clarification operation. Various studies have pointed out that clear apple juices have low nutritional density [74] and less powerful antioxidant potential due to clarification process. However, results from study carried out by Amobonye et al. [75] showed that polygalacturonase treatment of pear juice preserved antioxidant potential and phenolic content, unlike certain conventional juice treatments. Thermal treatment of raw juice (pasteurization) caused the continuation of the negative trend in the content of polyphenolic components. As Javdani et al. [76] stated, different pasteurization regimes (duration and temperature) inevitably cause losses of polyphenols. 

**Figure 1:**
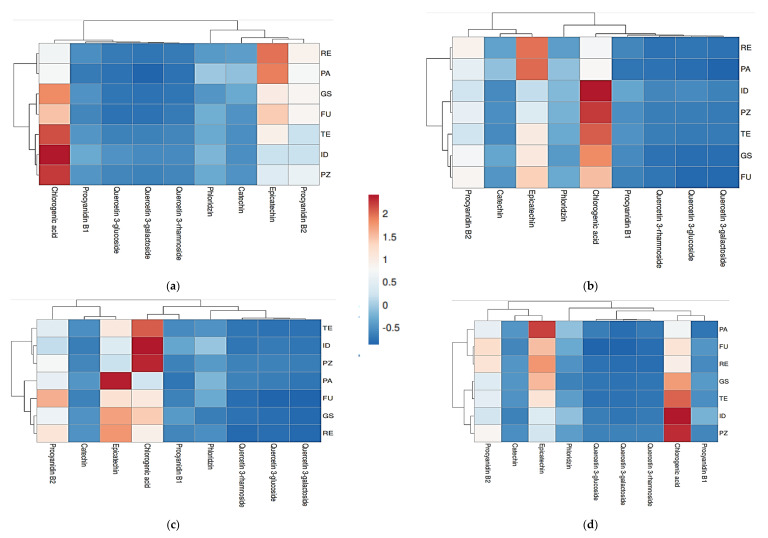
Heatmap of cloudy (**a**) and clear (**c**) juices without L-ascorbic acid and cloudy (**b**) and clear (**d**) juices with added L-ascorbic acid. Abbreviations: ID—‘Idared’, GS—‘Granny Smith’, PZ—‘Prijedorska zelenika’, FU—‘Funtača’, RE—‘Rebrača’, TE—‘Tetovka’, PA—‘Paradija’. Similarity of apple juices in polyphenolic compounds content is displayed by different colours which indicate lower (blue) and higher values (red).

The total losses of individual components from raw materials to cloudy juices without L-ascorbic acid varied from 82.9% (Prijedorska zelenika) to 90.9% (Idared), and in juices with L-ascorbic acid pretreatment from 79.0% (Funtača) to 90.8% (Granny Smith). When it comes to clear juices, longer exposure to oxygen and applied clarification operations resulted in higher degradation of polyphenols, which is in accordance with results reported by Hyson [77], Candrawinata et al. [78], and Koutsos et al. [79]. Chlorogenic acid, epicatechin and procyanidin B2 were the main polyphenolic compounds present in all the types of produced juices, as visible on heatmaps (Figure 1). These results are consistent to those reported by Dushkova et al. [80], Yang et al. [31], and Tian et al. [81].

## 3. Materials and Methods

### 3.1. Plant Material

In the present study, five traditional apple cultivars, ‘Paradija’ (PA), ‘Tetovka’ (TE), ‘Funtača’ (FU), ‘Prijedorska zelenika’ (PZ), and ‘Rebrača’ (RE), and two commercial apple cultivars, ‘Idared’ (ID) and ‘Granny Smith’ (GS), were used as a main raw material. According to Akagić and Vranac [34], Idared is the leading apple cultivar grown in Bosnia and Herzegovina due to its good storage capacity and predominant utilization in the fruit processing industry. Along with Idared, Granny Smith cultivar is often used. The selection of traditional apple cultivars was based on the previous studies [17,18,82] which showed that these cultivars are characterized by valuable technological, nutritional, bioactive, and sensory properties. Apples were cultivated in the ex situ collection in the orchard located in Srebrenik, North-East Bosnia, and Herzegovina (44°45′ N 18°28′ E; altitude 166 m). All accessions were grafted on MM106 rootstocks, planted 2 × 3.5 m apart. Standard commercial practice for integrated fruit production (pruning, spraying, irrigation, etc.) was followed. According to data from Federal Hydrometeorological Service (Sarajevo, B&H), average values of weather parameters during the apple growing season were the following: mean temperature 19.1 °C, insolation 258 h, cloudiness 3.85 C 0–8, precipitation 71 mm, 64.5 rainfall days.

An average of 50 kg per apple cultivar was harvested from trees at technological maturity stage (determined by iodine-starch test and by sensory evaluation of peel and pulp color). Immediately after harvest, apples were brought to the laboratory. Randomly chosen fruit samples per cultivar (30) were selected as representative sample for polyphenolic profiles analysis. Each fruit was peeled in order to separate peel and pulp samples, and cut into slices with a ceramic knife (~1 cm cutting depth), frozen in liquid nitrogen, and kept in polyethylene bags at −20 °C until analysis. The rest amounts of apples were used for juices production.

### 3.2. Prepartion of Apple Juices

The total amount of apples per each cultivar (cca 40 kg) was divided into four equal parts (4 × 10 kg) with the aim of production of four variants of monocultivar apple juices: (i) cloudy juice without L-ascorbic acid (C); (ii) cloudy juice with L-ascorbic acid (CAA); (iii) clear juice without L-ascorbic acid (CL); and (iv) clear juice with L-ascorbic acid (CLAA). Juice production was modified according to instructions given by Akagić [83] and was done in three repetitions. Table 4 shows operations, phases, and sampling points during juice production, as well as critical processes where the polyphenols’ loss was mostly expected.

After inspection and washing, apples were ground in a stainless steel mill. According to Krapfenbauer et al. [84], L-ascorbic acid is added to prevent oxidation in the amount of 150 mg kg^−1^. Required amounts of L-ascorbic acid for production of CAA and CLAA juices were added during the grinding of apples. By the end of this operation, apple mash samples were taken (Phase I). Mash enzymatization (30 min, without stirring, at room temperature) was done by using Fructozym MA (Erbslöh, Geisenheim, Germany). Apple mashes were pressed by a stainless steel hand presser. Extracted raw juices were sampled (Phase II) prior to pasteurization in case of cloudy juices production. Pasteurization was carried out at 78 °C for 2 min. Hot juices were filled into sterilized dark glass bottles (0.2 L) and cooled in lukewarm water (~40 °C). After the final cooling in cold water, cloudy juices were sampled (Phase IVa) and kept at −20 °C until analysis.

For production of clear juices, depectinization of raw juices was carried out by using Fructozym P (Erbslöh, Geisenheim, Germany), at 50 °C during 1 h. An alcohol test was used for qualitative estimation of pectins presence in depectinized juices. By the end of complete pectins hydrolysis, juices were sampled (Phase III-1). The clearing of depectinized juices was made by the addition of NaCalit bentonite, ErbiGel gelatine, and Klar-Sol 30 silica salt (Erbslöh, Geisenheim, Germany) at room temperature during 1 h. For filtration of juices, a plate filter pump (Rover Colombo 12, Padua, Italy) with 5 single-use filters (Rover 16, 20 × 20 cm, 0.9 μm) was used. Filtered juices were sampled (Phase III-2). Pasteurization, filling, cooling and sampling of clear juices (Phase IV b) were done using the same methods for cloudy juices described above.

### 3.3. Solvents and Reagents

Analytical standards of polyphenols were purchased as follows: chlorogenic and gallic acids, (+)-catechin, (−)-epicatechin, procyanidin B1, procyanidin B2, quercetin-3-*O*-glucoside, quercetin-3-*O*-rhamnoside, and quercetin-3-*O*-rutinoside were obtained from Fluka (Buchs, Switzerland), phloridzin, phloretin, caffeic, and sinapic acids from Merck (Darmstadt, Germany), while protocatechuic acid and quercetin-3-*O*-galactoside were from Sigma-Aldrich (Steinheim, Germany).

Methanol, acetonitrile (both of HPLC grade), formic acid and butylated hydroxytoluene (BHT) were sourced from Sigma-Aldrich (Steinheim, Germany).

### 3.4. Individual Polyphenolic Compounds Extraction and Analysis (RP-HPLC/DAD) of Apples and Juices

Apple materials (separately taken 5 g of peel and 10 g of pulp as fresh weight—FW) were homogenized with a 10 mL of extraction solution (methanol mixed with 3% *v*/*v* formic acid and 1% *m*/*v* butylated hydroxytoluene—BHT). Samples were ultrasonified in an ultrasonic ice bath (Elmasonic S 69 H; Elma Schmidbauer, Singen, Germany) for 1 h, followed by centrifuging at 10,000 rpm for 7 min at 0 °C (Thermo Scientific SL16 Centrifuge Series, San Jose, CA, USA). The obtained supernatant was filtered into vial through the Chromafil AO-45/25 polyamide filter (Macherey-Nagel, Düren, Germany).

Samples of apple juices were diluted, centrifuged, and filtered prior to analysis by the same procedure described above.

Individual polyphenolic compounds were analyzed using the Thermo Scientific Finnigan Surveyor HPLC-DAD system, controlled by a ChromQuest 4.0 chromatography workstation software system (Thermo Scientific, San Jose, CA, USA). Separation of polyphenolic compounds was achieved by using Pursuit XRs 3 C-18 column (4.6 × 150 mm, 5 µm; Agilent Technologies, Santa Clara, CA, USA) operated at 25 °C. The elution solvents were 97% acetonitrile + 3% bidistilled water + 0.1% formic acid (A), and 97% bidistilled water + 3% acetonitrile + 0.1% formic acid (B). The sample injection volume was 20 µL and a flow rate maintained at 0.6 mL min^−1^ during 45 min. The samples were eluted with the following gradient program: 0–15 min (5% A; 95% B), 15–20 min (20% A; 80% B), 20–30 min (30% A; 70% B), 30–35 min (90% A; 10% B), 35–45 min (0% A; 100% B), 45 min (5% A; 95% B); washing and reconditioning the column. The detection of polyphenolic compounds was carried out with a diode array detector (DAD). Phenolic acids (chlorogenic, caffeic, protocatechuic, gallic, and sinapic acid), flavan-3-ols ((+)-catechin, (−)-epicatechin, procyanidin B1, and procyanidin B2), and dihydrochalcones (phloridzin and phloretin) were analyzed at 280 nm, while flavonol 3-glycosides (quercetin-3-*O*-glucoside, quercetin-3-*O*-galactoside, quercetin 3-*O*-rhamnoside, and quercetin 3-*O*-rutinoside) were detected at 350 nm. Quantification of polyphenols was made according to retention times and corresponding external standard. The content of individual polyphenolic compounds was expressed in mg kg^−1^ of fresh weight (FW) for apples’ peel and pulp and mg L^−1^ for juices. Content of phenolic groups present in different parts of apples was calculated as percentage share (%) of these groups according to total content detected in the whole fruit (peel + pulp contents). Percentage loss (%) of polyphenols from raw material to intermediate and final products was calculated on the basis of difference in initial and recorded phenolic content values per each processing phase.

### 3.5. Statistical Data Analysis

Univariate statistical analysis included the calculation of means and standard deviations by using SPSS 20.0 statistical software (Chicago, IL, USA). Two-way factorial analysis of variance (ANOVA), followed by post hoc Tukey’s test at *p* ≤ 0.05 was used for testing the impact of cultivar (traditional and commercial) and part of fruit (peel and pulp) on each polyphenolic compound content in both apple peel and pulp. The same analysis was applied on testing the influence of cultivar and L-ascorbic addition on polyphenols content throughout different juice processing phases. The visual presentation of polyphenolic compounds, retained in four variants of final apple juices, was obtained by using a heatmap function (ClustVis program package. Available online: https://biit.cs.ut.ee/clustvis/online accessed on 22 October 2022). The heatmap shows clusters of both rows and columns, along with correlation distance and linkage. Similarity of apple juices made from different cultivars in polyphenolic compounds content is displayed by scale of colors which indicate lower (blue) and higher values (red).

## 4. Conclusions

Obtained results revealed that traditional apple cultivars are rich sources of polyphenols and they are of particular importance regarding their nutraceutical value. However, processing apples into juices has significant effect on the variation of the content and the type of polyphenols present in the final products. The grinding and pressing operations are crucial phases at which most changes occur in polyphenolic composition, due to oxidation process and component solubility. Loss of polyphenols is greater in production of clear juices where clarification occurs. The polyphenolic content decrease can be slightly reduced by L-ascorbic acid mash pretreatment, but not in a great extent. As a bottom line, it can be stated that cloudy apple juices made from traditional cultivars and with added L-ascorbic acid retain the most polyphenols and thus the highest antioxidant potential.

## Figures and Tables

**Table 1:** Average content of individual polyphenolic compounds in apple cultivars (mg kg^−1^ of FW) ± standard deviation (SD).

Phenolic Compounds	Idared		Granny Smith		P. zelenika		Funtača		Rebrača		Tetovka		Paradija	
	P	PU	P	PU	P	PU	P	PU	P	PU	P	PU	P	PU
**Phenolic acids**														
Chlorogenic acid	85.6 ± 1.5	136.4 ± 1.6	50.2 ± 0.8	71.8 ± 0.8	217.6 ± 2.5	389 ± 4.4	50 ± 2.6	142.1 ± 2.7	81.6 ± 0.99	117.3 ± 0.8	181.4 ± 3.5	130.1 ± 3.5	68.4 ± 1.3	101.2 ± 0.5
	111 ± 1.55 a*		61.0 ± 0.8 b*		303.3 ± 3.5 c*		96.1 ± 2.7 d*		99.5 ± 0.9 d*		155.8 ± 3.5 e*		84.8 ± 0.9 f*	
Caffeic acid	3.0 ± 0.2	1.1 ± 0.01	1.9 ± 0.2	0.3 ± 0.03	1.93 ± 0.07	1.06 ± 0.04	2.64 ± 0.2	0.52 ± 0.04	1.44 ± 0.09	0.37 ± 0.02	1.68 ± 0.14	0.39 ± 0.01	2.19 ± 0.29	1.46 ± 0.004
	2.10 ± 0.1 a*		1.10 ± 0.1 b*		1.50 ± 0.1 c*		1.58 ± 0.12 cd*		0.91 ± 0.11 b*		1.04 ± 0.08 b*		1.83 ± 0.1 ad*	
Gallic acid	8.5 ± 0.8	2.49 ± 0.14	9.36 ± 0.4	3.25 ± 0.06	6.74 ± 0.21	2.95 ± 0.04	4.1 ± 0.38	5.61 ± 0.39	5.85 ± 0.27	1.24 ± 0.03	5.12 ± 0.95	3.45 ± 0.34	6.43 ± 0.27	3.02 ± 0.24
	5.50 ± 0.5 a*		6.31 ± 0.2 b*		4.85 ± 0.1 ad*		4.86 ± 0.39 ad*		3.55 ± 0.15 c*		4.29 ± 0.6 cd*		4.73 ± 0.26 ad*	
Protocatechuic acid	5.87 ± 0.25	1.0 ± 0.02	6.28 ± 0.87	1.66 ± 0.19	6.76 ± 0.2	3.73 ± 0.17	1.27 ± 0.11	1.85 ± 0.13	7.52 ± 0.22	1.24 ± 0.09	4.85 ± 0.14	1.2 ± 0.04	4.83 ± 0.17	0.76 ± 0.01
	3.44 ± 0.1 a*		3.97 ± 0.5 b*		5.25 ± 0.2 c*		1.56 ± 0.12 d*		4.38 ± 0.2 b*		3.02 ± 0.1 ae*		2.80 ± 0.1 e*	
Sinapic acid	99.4 ± 1.2	0.4 ± 0.05	19.1 ± 2.8	0.01 ± 0.01	6.39 ± 0.1	1.16 ± 0.06	2.1 ± 0.1	0.42 ± 0.03	9.4 ± 0.7	0.8 ± 0.05	15.3 ± 0.5	1.23 ± 0.2	27.4 ± 1.0	1.23 ± 0.16
	49.9 ± 0.6 a*		9.56 ± 1.4 b*		3.78 ± 0.1 c*		1.26 ± 0.1 d*		5.10 ± 0.4 c*		8.27 ± 0.4 b*		14.3 ± 0.6 e*	
**Σ**	202.4	141.4	86.9	77.0	239.4	397.9	60.1	150.5	105.8	121.0	208.4	136.4	109.3	107.7
**Flavan 3-ols**														
Catechin	28.9 ± 0.3	1.82 ± 0.1	9.25 ± 0.3	22.9 ± 1.3	58.0 ± 1.6	5.36 ± 0.2	6.26 ± 0.8	16.3 ± 1.0	61.7 ± 2.8	29.5 ± 4.9	12.2 ± 1.2	36.3 ± 1.3	110.4 ± 0.4	68.8 ± 2.6
	15.4 ± 0.2 a*		16.1 ± 0.8 a*		31.7 ± 0.9 b*		11.3 ± 0.9 c*		45.6 ± 3.9 d*		24.3 ± 1.3 e*		89.6 ± 1.5 f*	
Epicatechin	74.8 ± 1.1	29.9 ± 1.1	88.1 ± 1.6	2.88 ± 0.1	95.2 ± 0.5	43.1 ± 0.6	152.3 ± 2.8	43.5 ± 3.8	244.3 ± 2.6	42.1 ± 1.97	194 ± 0.5	26.1 ± 0.8	302.1 ± 1.1	52.8 ± 0.1
	52.4 ± 1.1 a*		45.5 ± 0.9 b*		69.2 ± 0.6 c*		97.9 ± 3.3 d*		143.2 ± 2.3 e*		110.1 ± 0.7 f*		177.5 ± 0.6 g*	
Procyanidin B1	58.1 ± 0.8	16.9 ± 0.2	12.6 ± 1.2	0.66 ± 0.2	20.1 ± 0.3	12.4 ± 0.6	30.8 ± 1.4	9.88 ± 0.7	31.2 ± 2.3	4.09 ± 0.3	26.4 ± 0.5	7.65 ± 0.2	9.04 ± 0.1	13.3 ± 0.3
	37.5 ± 0.5 a*		6.63 ± 0.7 b*		16.3 ± 0.5 c*		20.3 ± 1.1 d*		17.6 ± 1.3 c*		17.0 ± 0.4 c*		11.2 ± 0.2 e*	
Procyanidin B2	87.2 ± 1.1	44.2 ± 0.3	101.3 ± 1.8	2.83 ± 0.1	113.4 ± 2.4	60.1 ± 2.6	120 ± 4.3	34.0 ± 3.7	149 ± 1.4	26.5 ± 1.1	108.4 ± 0.4	20.1 ± 1.2	146.6 ± 0.7	23.7 ± 0.6
	65.7 ± 0.7 a*		52.1 ± 1.0 b*		86.8 ± 2.5 c*		77.0 ± 4.0 d*		87.8 ± 1.3 c*		64.3 ± 0.8 a*		85.2 ± 0.7 c*	
**Σ**	249.0	92.8	211.3	29.3	286.7	121.0	309.4	103.7	486.2	102.2	341.0	90.2	568.1	158.6
**Dihydrochalcones**														
Phloridzin	73.5 ± 1.4	11.0 ± 1.3	36.2 ± 1.5	6.07 ± 0.9	82.3 ± 2.0	12.1 ± 0.6	58.5 ± 0.6	6.52 ± 0.3	83.9 ± 0.8	18.4 ± 0.3	169 ± 2.9	9.56 ± 1.5	169.7 ± 0.6	9.76 ± 0.8
	42.3 ± 1.4 a*		21.1 ± 1.2 b*		47.2 ± 1.3 c*		32.5 ± 0.5 d*		51.2 ± 0.6 e*		89.3 ± 4.4 f*		89.7 ± 0.7 f*	
Phloretin	8.79 ± 0.1	3.87 ± 0.1	24.8 ± 2.4	4.55 ± 0.4	20.0 ± 1.1	3.44 ± 0.1	11.1 ± 0.7	2.64 ± 0.1	25.0 ± 1.4	3.84 ± 0.2	26.7 ± 0.8	3.66 ± 0.17	48.5 ± 0.1	3.39 ± 0.1
	6.33 ± 0.1 a*		14.7 ± 3.0 b*		11.7 ± 0.6 c*		6.87 ± 0.3 a*		14.4 ± 0.8 b*		15.2 ± 0.5 b*		25.9 ± 0.1 d*	
**Σ**	82.3	14.9	61.0	10.6	102.3	15.5	69.6	9.16	108.9	22.2	195.7	13.2	218.2	13.2
**Flavonol glycosides**														
Quercetin 3-O-galactoside	174.2 ± 1.3	0.42 ± 0.05	32.7 ± 0.17	0.51 ± 0.01	51.3 ± 0.6	0.4 ± 0.02	9.21 ± 0.6	0.51 ± 0.04	58.1 ± 0.3	2.67 ± 0.4	20.2 ± 1.4	0.42 ± 0.07	38.1 ± 0.7	1.54 ± 0.2
	87.3 ± 0.7 a*		16.6 ± 0.1 b*		25.9 ± 0.3 c*		4.86 ± 0.3 d*		30.4 ± 0.4 e*		10.3 ± 0.7 f*		19.8 ± 0.5 g*	
Quercetin 3-O-glucoside	90.8 ± 0.5	0.36 ± 0.05	17.4 ± 0.9	0.11 ± 0.001	37.1 ± 1.14	0.18 ± 0.01	9.11 ± 0.8	0.17 ± 0.02	19.9 ± 1.9	0.19 ± 0.03	16.7 ± 0.7	0.19 ± 0.007	10.6 ± 0.2	0.54 ± 0.06
	45.6 ± 0.3 a*		8.76 ± 0.5 de*		18.6 ± 0.6 b*		4.64 ± 0.4 c*		10.0 ± 1.0 d*		8.45 ± 0.4 e*		5.57 ± 0.1 c*	
Quercetin 3-O-rhamnoside	111 ± 0.5	0.24 ± 0.005	41.4 ± 0.8	0.34 ± 0.03	36.8 ± 0.8	0.9 ± 0.03	21.1 ± 1.8	0.7 ± 0.05	69.7 ± 1.4	1.45 ± 0.2	47.3 ± 1.2	0.36 ± 0.06	88.1 ± 0.7	1.01 ± 0.001
	55.6 ± 0.3 a*		20.9 ± 0.4 b*		18.9 ± 0.4 c*		10.9 ± 0.9 d*		35.6 ± 0.8 e*		23.8 ± 0.6 f*		44.6 ± 0.4 g*	
Quercetin 3-O-rutinoside	15.5 ± 1.3	nd	2.79 ± 0.4	nd	0.94 ± 0.2	nd	0.39 ± 0.03	nd	3.13 ± 0.05	nd	0.95 ± 0.04	nd	0.28 ± 0.02	nd
	15.5 ± 1.3 a*		2.79 ± 0.4 b*		0.94 ± 0.01 c*		0.39 ± 0.03 c*		3.13 ± 0.05 b*		0.95 ± 0.04 c*		0.28 ± 0.02 c*	
**Σ**	391.5	1.02	94.3	0.96	126.1	1.48	39.8	1.38	150.8	4.31	85.2	0.97	137.1	3.09
**TOTAL**	925.2	250.1	453.5	117.9	754.4	535.9	478.9	264.7	851.7	249.7	830.3	240.8	1032.7	282.6
**TOTAL FRUIT (P+PU)**	**1175.3**		**571.4**		**1290.3**		**743.6**		**1101.4**		**1071.1**		**1315.3**	

Abbreviations: P—peel; PU—pulp; nd—not detected. Mean values per fruit ± standard deviation (SD) in rows marked with different letters (a–g) represent statistically significant difference between cultivars for each polyphenolic compound; an asterisk (*) indicates statistically significant differences in polyphenolic compounds content between the peel and pulp per each apple cultivar (Tukey’s test, *p* ≤ 0.05).

**Table 2:** Average share of phenolic groups in peel, pulp and whole fruit (%).

Phenolic Group	Idared			Granny Smith			P. zelenika			Funtača			Rebrača			Tetovka			Paradija		
	P	PU	F	P	PU	F	P	PU	F	P	PU	F	P	PU	F	P	PU	F	P	PU	F
Phenolic acids	17.2	12	29.2	15.2	13.4	28.6	18.6	30.8	49.4	8.10	20.2	28.3	9.60	11.0	20.6	19.5	12.7	32.2	8.30	8.20	16.5
Flavan 3-ols	21.2	7.90	29.1	37.0	5.10	42.1	22.2	9.40	31.6	41.6	13.9	55.5	44.1	9.30	53.4	31.8	8.40	40.2	43.2	12.1	55.3
Dihydrochalcones	7.0	1.27	8.3	10.7	1.90	12.6	7.90	1.20	9.10	9.40	1.20	10.6	10.0	1.90	11.9	18.3	1.20	19.5	16.6	1.0	17.6
Flavonol glycosides	33.3	0.09	33.4	16.5	0.16	16.7	9.80	0.10	9.90	5.40	0.19	5.60	13.7	0.40	14.1	8.0	0.10	8.10	10.4	0.23	10.6
TOTAL SHARE	78.7	21.3	-	79.4	20.6	-	58.5	41.5	-	64.5	35.5	-	77.4	22.6	-	77.6	22.4	-	78.5	21.5	-

Abbreviations: P—peel; PU—pulp; F—whole fruit.

**Table 3:** Distribution of polyphenols content throughout different phases of juice production (mg kg^−1^ for mash; mg L^−1^ for juices).

*Phases*	*Cultivars*	*Phenolic Acids*			*Flavan 3-ols*												*Dihydrochalcones*			*Flavonol Glycosides*								
		Chlorogenic a.			Catechin			Epicatechin			Procyan. B1			Procyan. B2			Phloridzin			Q-3-galactoside			Q-3-glucoside			Q-3-rhamn.		
		−	+		−	+		−	+		−	+		−	+		−	+		−	+		−	+		−	+	
PHASE I—MASH	ID	170.8 ± 0.6	183 ± 0.9	a*	18.8 ± 0	22.1 ± 0.5	a*	76.8 ± 0.3	78 ± 0.8	a*	45.7 ± 0.3	48.5 ± 1	a*	89.8 ± 0.1	104.4 ± 0.9	a*	45.3 ± 0	50.8 ± 0	a*	60.7 ± 0.1	65.2 ± 0.8	a*	30.6 ± 0.1	35.5 ± 0.8	a*	41.4 ± 1.2	43.7 ± 0.2	a*
	GS	76.3 ± 0.5	83.1 ± 1.6	b*	20.7 ± 0	26.6 ± 0.4	b*	56 ± 0.2	64.4 ± 0.3	b*	7.74 ± 0.2	9.19 ± 0	b*	50.2 ± 0.4	57.7 ± 0.4	b*	16.2 ± 1	18.9 ± 1	b*	9.7 ± 0.3	10.4 ± 0.1	b*	7.77 ± 0.2	8.82 ± 0.1	b*	17.2 ± 0.1	18.7 ± 0.1	b*
	PZ	329.7 ± 0.5	350.6 ± 0.1	c*	38.2 ± 1	44 ± 0.8	c*	105.6 ± 0.1	112 ± 0.8	c*	25.7 ± 0.4	26.8 ± 0	c*	123.6 ± 0.6	128.3 ± 0.8	c*	65.8 ± 0	71.1 ± 1	c*	18.8 ± 0.6	21.9 ± 0.6	c*	19.4 ± 0.2	22.4 ± 0.3	c*	24.2 ± 0.2	26.1 ± 0.3	c*
	FU	130.9 ± 0.2	148.5 ± 0.7	d*	45.1 ± 0	49.6 ± 0.3	d*	145.1 ± 0.5	153.5 ± 1.4	d*	11.98 ± 0.4	16.0 ± 0	d*	72.0 ± 0.4	81.8 ± 1.4	d*	23.9 ± 1	30.1 ± 1	d*	2.91 ± 0.1	3.99 ± 0.2	d*	2.35 ± 0.2	3.33 ± 0.1	d*	7.78 ± 0.3	9.51 ± 0.1	d*
	RE	110.5 ± 0.3	119.5 ± 0.9	e*	53.8 ± 1	60.9 ± 0.4	e*	225.9 ± 1.1	234.4 ± 0.4	e*	17.7 ± 0.8	20.5 ± 0	e*	113.4 ± 0.8	118.5 ± 0.8	e*	45.7 ± 1	54.6 ± 0	e*	28.5 ± 0.3	33.2 ± 0.5	e*	12.1 ± 0.2	13.0 ± 0.2	e*	32.4 ± 0.9	36.6 ± 0.5	e*
	TE	245.6 ± 2.8	271.9 ± 1.0	f*	26.7 ± 1	31.1 ± 0.6	f*	182.5 ± 0.5	191.9 ± 0.5	f*	25.8 ± 0.7	27.5 ± 1	c*	102.8 ± 0.5	109.9 ± 0.8	f*	74.7 ± 1	92.2 ± 1	f*	7.4 ± 0.2	8.68 ± 0.1	f*	8.95 ± 0.1	10.3 ± 0.1	f*	18.6 ± 0.3	22.6 ± 0.5	f*
	PA	152.1 ± 0.1	160.9 ± 0.3	g*	126 ± 0	137.1 ± 2	g*	217 ± 1.6	234 ± 0.8	g*	16.0 ± 0.5	17.2 ± 0	f*	125.5 ± 0.9	131.3 ± 0.6	g*	72.7 ± 1	79.2 ± 1	g*	15.6 ± 0.3	18.1 ± 0.1	g*	5.52 ± 0.1	6.82 ± 0.1	g*	22.1 ± 0.4	26.4 ± 1.5	c*
PHASE II—RAW JUICE	ID	138.7 ± 0.9	147.3 ± 0.4	a*	12.4 ± 0	15.9 ± 0.1	a*	46.2 ± 0.7	49.5 ± 0.2	a*	26.6 ± 0.6	33.4 ± 1	a*	50.6 ± 0.2	60.3 ± 0.1	a*	36.2 ± 0	41.7 ± 1	a*	24.1 ± 0.1	26.8 ± 0.8	a*	18.5 ± 0.2	21.4 ± 0.5	a*	12.8 ± 0.1	15.1 ± 0.1	a*
	GS	50.1 ± 1.2	59.2 ± 0.1	b*	11.8 ± 0	16.5 ± 0.3	a*	42.4 ± 0.2	46.4 ± 0.1	b*	6.4 ± 0.03	6.86 ± 1	b*	34.6 ± 0.2	38.3 ± 0.4	b*	9.71 ± 0	13.5 ± 0	b*	1.08 ± 0.1	1.19 ± 0.1	b*	3.06 ± 0.1	3.82 ± 0.2	df*	3.1 ± 0.04	3.51 ± 0.1	b*
	PZ	277.7 ± 1.4	302.7 ± 1.5	c*	24.5 ± 0	30.1 ± 1.4	b*	80.9 ± 0.4	86.7 ± 0.2	c*	18.7 ± 0.1	19.6 ± 0	c*	96.5 ± 0.7	108.9 ± 0.6	c*	39.5 ± 1	46.9 ± 1	c*	12.6 ± 0.3	14.6 ± 0.1	c*	12.3 ± 0.5	14.5 ± 0.1	b*	18.4 ± 0.5	20.0 ± 0.2	c*
	FU	83.1 ± 0.9	100.4 ± 0.8	d*	19.1 ± 1	24.6 ± 0.5	c*	104 ± 1.0	115.3 ± 0.6	d*	9.68 ± 0.3	11.7 ± 0	d*	64.7 ± 0.8	70.5 ± 0.1	d*	21.2 ± 1	24 ± 1.0	d*	1.03 ± 0.1	1.26 ± 0.1	d*	1.18 ± 0.1	1.45 ± 0.1	c*	4.52 ± 0.1	6.08 ± 0.1	d*
	RE	81.2 ± 0.5	93.1 ± 0.4	e*	36.2 ± 1	39.4 ± 0.4	d*	148.4 ± 0.5	160.2 ± 0.8	e*	12.8 ± 0.7	16.0 ± 1	e*	91.6 ± 0.8	97.5 ± 0.7	e*	25.5 ± 0	33.7 ± 0	e*	7.58 ± 0.1	9.64 ± 0.2	b*	3.33 ± 0.1	3.92 ± 0.1	d*	5.35 ± 0.1	6.65 ± 0.1	e*
	TE	217.0 ± 2.5	241.7 ± 0.8	f*	14.0 ± 0	18.2 ± 0.4	e*	145.6 ± 0.3	162.1 ± 0.4	e*	18.9 ± 0.3	21.6 ± 1	f*	83.2 ± 0.5	95.4 ± 0.2	f*	45.1 ± 1	53.0 ± 0	f*	1.61 ± 0.1	2.50 ± 0.2	f*	2.04 ± 0.1	2.65 ± 0.2	e*	4.82 ± 0.1	6.48 ± 0.3	de*
	PA	111.5 ± 0.4	121.5 ± 0.4	g*	48.4 ± 1	58.6 ± 0.4	f*	169.2 ± 1.0	199.1 ± 0.1	f*	10.2 ± 0.5	11.5 ± 0	d*	80.6 ± 0.6	91.6 ± 1.0	g*	51.8 ± 1	59.8 ± 1	g*	10.1 ± 0.3	11.4 ± 0.3	b*	2.6 ± 0.1	3.65 ± 0.3	f*	10.8 ± 0.2	14.8 ± 0.3	f*
PHASE III 1—DEPECTINISATION	ID	99.5 ± 0.2	112.6 ± 0.3	a*	10.3 ± 0	12 ± 0.1	a*	38.7 ± 0.3	45 ± 0.4	a*	21.4 ± 0.3	27.3 ± 0	a*	33.9 ± 0.9	40 ± 0.2	a*	24.2 ± 0	28.6 ± 0	a*	3.04 ± 0.1	3.89 ± 0.1	a*	1.59 ± 0.1	1.85 ± 0.1	a*	3.18 ± 0.1	3.75 ± 0.2	a*
	GS	35.1 ± 0.6	40.6 ± 0.2	b*	4.11 ± 0	5.35 ± 0.1	b*	27.3 ± 0.6	32.07 ± 0.2	b*	4.65 ± 0.1	5.47 ± 0	b*	15.5 ± 0.1	19.9 ± 0.2	b*	7.68 ± 0	9.18 ± 0	b*	0.72 ± 0.1	0.87 ± 0.1	b*	0.6 ± 0.04	0.7 ± 0.003	b*	1.89 ± 0.1	2.28 ± 0.1	b*
	PZ	220.2 ± 0.6	254.4 ± 2.5	c*	22.9 ± 1	27.8 ± 0.4	c*	76.3 ± 0.6	88.5 ± 0.5	c*	15.4 ± 0.5	18.0 ± 1	c*	106.7 ± 0.5	115.0 ± 0.6	c*	40.3 ± 0	46.1 ± 0	c*	9.77 ± 0.2	11.6 ± 0.3	c*	5.72 ± 0.3	5.04 ± 1.8	c*	11.6 ± 0.3	13.6 ± 0.5	c*
	FU	71.6 ± 1	75.7 ± 0.6	d*	15.5 ± 1	18.2 ± 0.2	d*	82.0 ± 0.5	89.5 ± 1.0	d*	13.3 ± 0.6	17.8 ± 0	d*	56.6 ± 0.1	59.9 ± 0.2	d*	16.3 ± 1	19.8 ± 0	d*	0.51 ± 0.1	0.68 ± 0.1	d*	0.37 ± 0.1	0.49 ± 0.1	b*	2.92 ± 0.1	3.48 ± 0.1	d*
	RE	121.3 ± 0.4	137.4 ± 0.5	e*	28.4 ± 0	33.9 ± 0.8	e*	210.9 ± 0.6	221.5 ± 0.8	e*	18.0 ± 0.4	19.9 ± 1	e*	128.3 ± 0.3	140 ± 0.8	e*	29.9 ± 1	36.7 ± 1	e*	5.09 ± 0.1	6.07 ± 0.1	e*	2.54 ± 0.3	2.99 ± 0.4	d*	3.29 ± 0.1	4.03 ± 0.1	e*
	TE	196.3 ± 0.3	218.9 ± 0.6	f*	12.4 ± 0	14.6 ± 0.1	f*	114.4 ± 0.9	133.2 ± 0.4	f*	14.0 ± 0.4	15.1 ± 0	f*	60.2 ± 0.5	68.1 ± 0.7	f*	20.1 ± 1	25.5 ± 0	f*	1.15 ± 0.1	1.27 ± 0.1	f*	1.60 ± 0.1	1.68 ± 0.1	a*	3.20 ± 0.5	3.63 ± 0.7	a*
	PA	115.8 ± 0.7	129.5 ± 0.5	g*	52.7 ± 1	57.2 ± 1.6	g*	235.5 ± 0.8	253.8 ± 2.3	g*	9.52 ± 0.4	11.7 ± 0	g*	107.8 ± 0.7	117.8 ± 0.7	g*	79.5 ± 1	83.8 ± 1	g*	12.5 ± 0.4	14.6 ± 0.5	g*	1.78 ± 0.1	2.53 ± 0.1	ad*	10.8 ± 0.1	11.3 ± 0.1	f*
PHASE III 2—CLARIFICATION	ID	84.4 ± 0.7	96.9 ± 0.5	a*	7.05 ± 0	9.47 ± 0.1	a*	38.8 ± 0.5	34.4 ± 0.1	a*	14.6 ± 0.8	21.9 ± 0	a*	25.4 ± 0.5	31.3 ± 0.6	a*	18.6 ± 0	20.2 ± 0	a*	2.88 ± 0.1	2.91 ± 0.1	a*	1 ± 0.002	1.17 ± 0.1	a*	2.31 ± 0.2	2.52 ± 0.3	ae*
	GS	22.8 ± 0.5	32.2 ± 0.5	b*	3.38 ± 0	3.57 ± 0.1	b*	21.3 ± 0.3	27.5 ± 0.3	b*	4.42 ± 0.1	5.22 ± 0	b*	12.5 ± 0.4	14.6 ± 0.2	b*	3.77 ± 1	4.93 ± 0	b*	0.51 ± 0.1	0.72 ± 0.3	ce*	0.41 ± 0.1	0.52 ± 0.1	b*	1.43 ± 0.3	1.66 ± 0.1	b*
	PZ	171.9 ± 1.4	197.3 ± 1.1	c*	7.74 ± 1	11.5 ± 0.5	c*	42.5 ± 0.9	50.3 ± 0.2	c*	6.57 ± 0.5	8.70 ± 1	c*	69.5 ± 1.1	81.2 ± 1.1	c*	15.6 ± 1	17.6 ± 0	c*	5.67 ± 0.5	8.07 ± 0.1	b*	2.53 ± 0.4	3.46 ± 0.3	c*	5.35 ± 0.6	6.14 ± 0.1	c*
	FU	58.9 ± 0.4	63.4 ± 0.4	d*	10.1 ± 0	11.5 ± 0.4	d*	58.2 ± 0.9	68.4 ± 0.7	d*	8.56 ± 1.4	13.1 ± 1	d*	49.9 ± 0.6	54.2 ± 0.2	d*	14.5 ± 0	16.4 ± 0	d*	0.3 ± 0.1	0.35 ± 0.1	c*	0.22 ± 0.2	0.30 ± 0.1	b*	1.82 ± 0.1	2.41 ± 0.1	a*
	RE	102.3 ± 1.1	113.3 ± 1.5	e*	18.9 ± 1	23.3 ± 0.6	e*	169.4 ± 1.5	180.9 ± 0.5	e*	15.3 ± 0.7	16.1 ± 1	e*	112.7 ± 1.9	118.7 ± 1.0	e*	17.0 ± 1	20.1 ± 1	a*	3.22 ± 0.2	4.04 ± 0.1	d*	1.66 ± 0.1	2.10 ± 0.1	d*	1.68 ± 0.2	2.56 ± 0.1	a*
	TE	159.4 ± 1.3	175.7 ± 0.5	f*	9.43 ± 0	14.5 ± 0.4	f*	86.2 ± 1.0	100.5 ± 1.0	f*	10.8 ± 0.2	11.8 ± 0	d*	54.4 ± 0.7	60.8 ± 0.5	f*	14.9 ± 0	19.0 ± 0	c*	0.72 ± 0.1	0.85 ± 0.4	e*	0.85 ± 0.3	1.33 ± 0.2	a*	2.6 ± 0.004	2.87 ± 0.3	e*
	PA	50.4 ± 1.0	70.2 ± 1.1	d*	16.6 ± 1	21.8 ± 1.1	g*	119.3 ± 1.3	149.8 ± 2.2	g*	3.51 ± 0.4	4.73 ± 1	b*	58.4 ± 1.1	66.1 ± 1.8	g*	26.7 ± 1	45.4 ± 1	e*	6.56 ± 0.4	10.7 ± 0.2	f*	1.2 ± 0.003	2.0 ± 0.03	d*	5.37 ± 0.3	6.93 ± 0.1	f*
PHASE IVa—CLOUDY JUICE	ID	113.5 ± 0.4	132.4 ± 0.6	a*	4.56 ± 0	5.58 ± 0.1	a*	31.3 ± 0.1	36.6 ± 1	a*	12.8 ± 0.7	15.6 ± 0	a*	30.9 ± 0.3	39.9 ± 0.6	a*	16.3 ± 0	22.6 ± 0	a*	5.3 ± 0.05	5.7 ± 0.004	a*	3.17 ± 0.1	4 ± 0.1	a*	3.68 ± 0.3	4.27 ± 0.4	a*
	GS	32.5 ± 0.4	38.6 ± 0.5	b*	6 ± 0.4	7.28 ± 0.5	b*	21.9 ± 0.6	27.4 ± 0.4	b*	3.65 ± 0.1	4.26 ± 0	b*	20.1 ± 0.4	22.4 ± 0.5	b*	4.2 ± 0	5.77 ± 0	b*	0.77 ± 0.1	0.9 ± 0.03	b*	0.44 ± 0.1	0.59 ± 0.1	b*	0.75 ± 0.1	1.15 ± 0.1	b*
	PZ	177.2 ± 0.5	203.9 ± 1.1	c*	10.9 ± 0	14.3 ± 0.3	c*	68.2 ± 1.3	77.4 ± 0.2	c*	13.3 ± 0.1	14.9 ± 0	a*	77.5 ± 0.7	88.5 ± 0.4	c*	23.0 ± 0	33.6 ± 1	c*	4.56 ± 0.3	5.76 ± 0.3	c*	2.93 ± 0.1	4.09 ± 0.1	a*	4.95 ± 0.4	6.6 ± 0.6	c*
	FU	69.1 ± 0.4	82 ± 0.5	d*	9.2 ± 0	12.8 ± 0.2	d*	67 ± 0.3	77.3 ± 0.9	d*	5.49 ± 0.2	7.25 ± 0	c*	49 ± 0.5	58.6 ± 0.9	d*	15.5 ± 1	18.8 ± 0	d*	0.62 ± 0.1	0.73 ± 0.2	b*	0.5 ± 0.01	0.63 ± 0.1	b*	2.62 ± 0.1	3.43 ± 0.1	d*
	RE	68.8 ± 0.9	77.2 ± 0.6	e*	17.1 ± 0	23.7 ± 0.3	e*	128.9 ± 0.3	139 ± 0.4	e*	10.2 ± 0.2	11.2 ± 0	d*	78.2 ± 0.8	84.8 ± 0.7	e*	15.4 ± 1	21.4 ± 0	a*	2.94 ± 0.1	4.63 ± 0.1	d*	1.75 ± 0.4	2.74 ± 0.2	c*	2.91 ± 0.1	3.49 ± 0.3	d*
	TE	196.3 ± 0.5	208.5 ± 0.6	f*	8.36 ± 0	10.5 ± 0.8	f*	111.7 ± 1.5	130.9 ± 0.6	f*	12.0 ± 0.2	15.3 ± 1	a*	60.5 ± 0.7	70.9 ± 0.8	f*	25.5 ± 1	31.3 ± 1	c*	1.40 ± 0.1	1.57 ± 0.1	e*	1.62 ± 0.1	1.69 ± 0.1	d*	4.01 ± 0.1	4.30 ± 0.4	a*
	PA	70.6 ± 0.3	87.3 ± 0.1	g*	34.2 ± 0	41.6 ± 0.3	g*	119.7 ± 1	151 ± 1.6	g*	9.03 ± 0.3	9.67 ± 0	e*	70.2 ± 0.2	74.1 ± 0.8	g*	36.8 ± 0	42.0 ± 1	e*	5.79 ± 0.1	7.35 ± 0.1	f*	1.56 ± 0.2	2.77 ± 0.2	c*	5.84 ± 0.1	8.35 ± 0.3	f*
PHASE IVb—CLEAR JUICE	ID	67.9 ± 0.5	81.8 ± 1.2	a*	2.33 ± 0	3.1 ± 0.03	a*	25 ± 0.3	29.5 ± 0.7	a*	9.05 ± 0.2	13.2 ± 1	a*	19.6 ± 0.4	26.4 ± 0.1	a*	15.2 ± 0	16.9 ± 0	a*	1.84 ± 0.1	2.35 ± 0.1	a*	0.79 ± 0.3	1.02 ± 0.3	a*	1.2 ± 0.001	1.36 ± 0.3	a*
	GS	13.5 ± 0.5	20.1 ± 0.3	b*	2.04 ± 0	2.66 ± 0.1	a*	15.4 ± 0.3	18.6 ± 0.1	b*	2.51 ± 0.1	2.98 ± 0	b*	8.9 ± 0.08	9.67 ± 0.1	b*	1.11 ± 0	1.37 ± 0	b*	0.34 ± 0.4	0.47 ± 0.1	cd*	0.11 ± 0.1	0.28 ± 0.1	b*	0.5 ± 0.001	0.6 ± 0.004	b*
	PZ	121.8 ± 1.7	131.4 ± 0.9	c*	4.25 ± 0	6.91 ± 0.1	b*	36.0 ± 2.2	41.0 ± 0.5	c*	3.04 ± 0.1	4.12 ± 0	c*	57.8 ± 2.0	66.8 ± 0.8	c*	10.5 ± 0	12.5 ± 0	c*	2.39 ± 0.3	3.05 ± 0.4	b*	1.71 ± 0.2	2.10 ± 0.1	c*	2.48 ± 0.2	3.09 ± 0.3	c*
	FU	36.2 ± 0.2	46.5 ± 0.5	d*	4.72 ± 0	6.71 ± 0.1	b*	38.8 ± 1.0	54.6 ± 0.5	d*	4.98 ± 0.4	9.23 ± 1	d*	46.7 ± 0.5	49.1 ± 0.8	d*	10.6 ± 0	13.4 ± 0	c*	0.09 ± 0.1	0.15 ± 0.1	c*	0.18 ± 0.1	0.2 ± 0.004	b*	1.60 ± 0.1	1.87 ± 0.1	d*
	RE	79.7 ± 0.9	85.8 ± 0.9	e*	15.1 ± 0	18.0 ± 0.5	c*	119.7 ± 2.1	124.1 ± 1.0	e*	11.0 ± 0.5	12.8 ± 0	e*	88.8 ± 0.7	95.1 ± 1.3	e*	14.1 ± 0	16.6 ± 1	a*	2.06 ± 0.1	3.04 ± 0.1	b*	1.01 ± 0	1.39 ± 0.1	d*	1.27 ± 0.2	2.0 ± 0.01	d*
	TE	103.2 ± 1.1	113.5 ± 1.0	f*	8.95 ± 0	11.5 ± 0.1	d*	66.2 ± 0.4	77.1 ± 1.2	f*	8.04 ± 0.3	9.31 ± 0	f*	44.3 ± 0.7	51.0 ± 0.3	d*	10.1 ± 1	14.4 ± 0	c*	0.48 ± 0.1	0.57 ± 0.1	d*	0.83 ± 0.1	0.98 ± 0.1	a*	1.66 ± 0.1	2.29 ± 0.1	e*
	PA	31.6 ± 1.7	55.8 ± 0.6	g*	9.44 ± 1	13.0 ± 0.5	e*	99.8 ± 1.8	113.3 ± 1.1	g*	1.52 ± 0.5	3.0 ± 0	b*	42.3 ± 1.7	51.2 ± 0.7	d*	18.3 ± 1	26.7 ± 1	d*	3.13 ± 0.1	5.17 ± 0.6	e*	0.85 ± 0.1	1.34 ± 0.1	d*	3.94 ± 0.1	4.36 ± 0.1	f*

Abbreviations: ID—‘Idared’, GS—‘Granny Smith’, PZ—‘Prijedorska zelenika’, FU—‘Funtača’, RE—‘Rebrača’, TE—‘Tetovka’, PA—‘Paradija’. ‘−’ stands for intermediate products and juices made without the addition of L-ascorbic acid, while ‘+’ indicates its presence. Results as mean ± standard deviation (SD) from three repetitions. Different letters (a–g) in columns per each phase represent statistically significant difference between cultivars in a polyphenolic compound content; an asterisk (*) indicates statistically significant difference in polyphenolic compound content between products with or without added L-ascorbic acid per each apple cultivar (Tukey’s test, *p* ≤ 0.05).

**Table 4:** Operations during the apple juices production.

Operations	C	CAA	CL	CLAA	Phase & Sampling Point	Critical Process
Inspection and washing	+	+	+	+		
Grinding	+	+	+	+	I	Cell wall disintegration
Grinding + addition of L-ascorbic acid		+		+		
Mash enzymatization	+	+	+	+	II	Extraction
Mash pressing	+	+	+	+		
Raw juice depectinization			+	+	III-1	Colloidal particles removal
Clarification			+	+	III-2	
Filtration			+	+		
Pasteurization	+	+	+	+	IV a/b*	Thermal treatment
Filling and cooling	+	+	+	+		

* ‘+’ indicates performed operation; ‘a’ is for cloudy juice, ‘b’ is for clear juice.

## Data Availability

The data supporting the reported results are available at request from the corresponding author.
